# Application and Validation of PFGE for Serovar Identification of *Leptospira* Clinical Isolates

**DOI:** 10.1371/journal.pntd.0000824

**Published:** 2010-09-14

**Authors:** Renee L. Galloway, Paul N. Levett

**Affiliations:** 1 Bacterial Zoonoses Branch, Centers for Disease Control and Prevention, Atlanta, Georgia, United States of America; 2 Saskatchewan Disease Control Laboratory, Regina, Saskatchewan, Canada; University of Washington, United States of America

## Abstract

Serovar identification of clinical isolates of *Leptospira* is generally not performed on a routine basis, yet the identity of an infecting serovar is valuable from both epidemiologic and public health standpoints. Only a small number of reference laboratories worldwide have the capability to perform the cross agglutinin absorption test (CAAT), the reference method for serovar identification. Pulsed-field gel electrophoresis (PFGE) is an alternative method to CAAT that facilitates rapid identification of leptospires to the serovar level. We employed PFGE to evaluate 175 isolates obtained from humans and animals submitted to the Centers for Disease Control and Prevention (CDC) between 1993 and 2007. PFGE patterns for each isolate were generated using the NotI restriction enzyme and compared to a reference database consisting of more than 200 reference strains. Of the 175 clinical isolates evaluated, 136 (78%) were identified to the serovar level by the database, and an additional 27 isolates (15%) have been identified as probable new serovars. The remaining isolates yet to be identified are either not represented in the database or require further study to determine whether or not they also represent new serovars. PFGE proved to be a useful tool for serovar identification of clinical isolates of known serovars from different geographic regions and a variety of different hosts and for recognizing potential new serovars.

## Author Summary

Leptospirosis is an infection caused by *Leptospira* bacteria, and is probably the most widespread zoonosis in the world. It is carried by a wide range of animals that contaminate the environment by shedding organisms in their urine. Humans become infected when they come into contact with contaminated urine or water in the environment that has been contaminated with the urine of infected animals. Despite its ubiquity, isolates are rarely identified to the serovar level due to the cumbersome, complicated serological methods that are involved. Serovar identification is important for epidemiology and enabling public health interventions. In this study, we employed a molecular method of serovar identification using pulsed-field gel electrophoresis to identify 175 clinical isolates of *Leptospira*. In order to validate this method for serovar identification, we also performed complex serological testing on a subset of the isolates. The results indicated that pulsed-field gel electrophoresis is an appropriate alternative to serological tests for serovar identification. Serovar identities of the clinical isolates are also discussed. Fifteen percent of the clinical isolates were identified as potentially new serovars and demonstrates the utility of a more rapid, standardized molecular method in order to keep up with the changing taxonomy and epidemiology of *Leptospira*.

## Introduction

Leptospirosis is a zoonotic infection found all over the world.[1] There is a wide range of animal hosts that maintain *Leptospira* organisms in their renal tubules and contaminate the environment.[2] Human cases usually occur due to contact with water or other environmental sources that have been contaminated with the urine of infected animals. Human cases can be severe and may cause multi-organ failure in previously healthy individuals.[3], [4]

The genus *Leptospira* is divided into 20 species, of which fourteen contain pathogenic and intermediately-pathogenic strains.[5], [6] Currently there are more than 250 pathogenic serovars organized into 24 serogroups based on antigenic relatedness.[7]–[10] Serovar identification of clinical isolates of *Leptospira* is important for understanding the epidemiology of leptospirosis. It can lead to the recognition of carrier mammals and enable targeted prevention methods in order to contain outbreaks, and it is important in identifying new species or serovars. However, serovar identification is not routinely performed in laboratories due to the difficulties involved in performing the cross agglutinin absorption test (CAAT), which is considered the reference method for serovar identification. The CAAT method requires the maintenance of large panels of reference antisera and live antigens, is time-consuming, and requires laboratory expertise to perform.[11] PFGE is an alternative method for the identification of *Leptospira* serovars;[12]–[15] however it has not been validated in the identification of clinical isolates. PFGE is quicker and easier to perform than CAAT, and digital analysis makes standardization and interpretation more accurate. PFGE has the added capability of differentiating between strains of serovars that belong to different species, whereas CAAT is unable to distinguish species differences in serovars such as Grippotyphosa, which appear in more than one species.[12], [16] PFGE is also able to rapidly highlight isolates that may represent new species or serovars, which makes it a very useful tool for taxonomic purposes.[12] In this study, we present the results of serovar identification of clinical isolates obtained from both human and animal sources worldwide and validate the use of PFGE for serovar identification using CAAT.

## Methods

*Leptospira* isolates from humans and animals were submitted for routine testing to the CDC between the years 2000 and 2007 from eight different countries for serovar identification. Two isolates received in 1993 and 1998 respectively were also included. A total of 175 isolates were analyzed by PFGE; a subset consisting of 36 isolates were also tested by CAAT to validate the PFGE method. Multilocus sequence typing (MLST) was also performed on 42 of the isolates as an additional molecular characterization method.

PFGE was performed using the NotI restriction enzyme to generate fingerprint patterns as previously described[12] using *Salmonella* Braenderup H9812 as a size standard.[17] Fingerprint patterns were analyzed using BioNumerics software (Applied Maths, Inc., Austin, TX). Dendrograms were created by UPGMA cluster analyses based on the Dice band-based coefficient. Band comparison settings of 1.5% optimization and 1% position tolerance were used. Fingerprint patterns of clinical isolates were queried against a library of >200 reference serovars (available to the public upon request) based on mean similarity. Those with fingerprint patterns matching a reference pattern in the library were identified to the serovar level. Serovars Icterohaemorrhagiae and Copenhageni are similar both serologically and genetically,[7], [18] and are also similar by PFGE.[13], [15] Therefore, they cannot be distinguished from one another using PFGE and will be referred to collectively in figures and tables as serovar Icterohaemorrhagiae.

MLST was performed on seven housekeeping genes,[19] and sequence types (STs) were determined from the resulting allelic profiles and compared to an established internet database (http://leptospira.mlst.net/). The current MLST scheme is only appropriate for two of the 14 pathogenic and intermediately-pathogenic species (*L. interrogans* and *L. kirschneri*); therefore MLST was not applicable to many isolates in this study (18% [30/170] of all isolates where the species was known), particularly to the potentially new serovars (not applicable to 39% [15/38]).

CAAT was performed as previously described.[11], [20] Briefly, the standard method using microscopic agglutination testing (MAT) was initially performed to determine serogroup classification using a panel of reference sera representing all pathogenic serogroups. Cross agglutinin absorption tests were then carried out using live reference strains that were serologically related to the unknown strain and sera were absorbed overnight. The absorbed sera were then tested using MAT. If the resulting titration using absorbed sera against the unknown strain gave a titer that was less than 10% of the homologous titer, the unknown strain was considered to belong to the same serovar as the reference strain.[20] Strains that could not be identified by cross agglutinin tests were designated for inoculation into rabbits to produce hyperimmune antisera and are currently undergoing serologic characterization.

16S rRNA gene sequencing was performed as previously described on nearly full-length 16S rRNA gene sequences.[21]

DNA relatedness and percentage divergence between strains were determined by the hydroxyapatite method[16], with 55°C used for optimal reassociation. The G + C content (mol%) was determined by the thermal denaturation method.[22] Samples were run at least three times, using DNA from *Escherichia coli* K-12 as a control.

## Results

Fingerprint patterns were generated for 175 clinical isolates of *Leptospira* from eight different countries. Isolates were obtained from humans, rodents/marsupials, and domestic animals (Table 1). The PFGE reference library identified 78% (136/175) of the isolates to the serovar level. An additional 15% (27/175) are being investigated further and were tentatively classified as new serovars. The remaining isolates (7%, 12/175) each may not be represented in the PFGE database, or may also represent new serovars and require further analysis. They have yet to undergo further studies as there is currently only one isolate found for each of these. The entire data set of PFGE results is represented in a dendrogram in Figure S1.

**Table 1:** Serovar identification results and CAAT results of isolates sent to CDC.^1^

Origin	Source (Number)	Serovar Identification (PFGE)	Number of Isolates	Confirmed by CAAT	MLST
Brazil	Dog (11), Human (2), Swine (2), Cow (1)	Canicola	16	5	3 (ST37,Pomona or Canicola)
	Human (13), Dog (1), Cow (1)	Icterohaemorrhagiae	15		
	Swine	Kennewicki^2^ (Pomona)	1		
	Rat	Biflexa	1		NA^3^
	Capybara	Unknown	6		NA
	Cow	Unknown	1		
	Capybara	Unknown	1		
**Total**			**41**		
United States - Hawaii	Human	Icterohaemorrhagiae	16	3	8 (ST17, Copenhageni or Icterohaemorrhagiae)
	Human	Ballum	4		NA
	Human	Unknown	17		12 (ST51, Australis)
	Human	Unknown	1		NA
	Human	Unknown	1		NA
	Human	Unknown	2		NA
United States - Other	Human	Icterohaemorrhagiae	1		
	Dog	Grippotyphosa (*L. kirschneri*)	1		
	Human	Unknown	2		NA
	Human	Unknown	2		NA
**Total**			**47**		
Egypt	Human	Icterohaemorrhagiae	12	3	3 (ST17, Copenhageni or Icterohaemorrhagiae)
	Human	Pomona	7	4	1 (ST37, Pomona or Canicola)
	Human	Bataviae	6	5	5 (ST50, Bataviae)
	Human	Pyrogenes	3	3	3 (ST88, 1 of 4 Pyrogenes ST types)
	Human	Grippotyphosa (*L. interrogans*)	3	2	1 (ST111, 1 of 4 Grippotyphosa ST types)
**Total**			**31**		
Peru	Rat (9), Spiny Rat (1)	Icterohaemorrhagiae	10	2	2 (ST17, Copenhageni or Icterohaemorrhagiae)
	Rat (7), Human (2)	*L. licerasiae*	9		NA
	Opossum	Unknown	1		
	Spiny Rat	Unknown	1		NA
	Opossum	Unknown	1		
**Total**			**22**		
Thailand	Human (24), Rat (3)	Bulgarica (*L.interrogans*)	27	7	1 (ST34, no reference match)
	Rat	Bataviae	1		
	Human	Unknown	1		1(ST113, no reference match)
	Human	Unknown	1		
	Rat	Unknown	1		
**Total**			**31**		
Denmark	Human	*L. broomii*	1		NA
France	Human	*L. broomii*	1		NA
Guyana	Human	Icterohaemorrhagiae	1	1	1 (ST17, Copenhageni or Icterohaemorrhagiae)

1 Only a subset of isolates were validated by CAAT; concordance between PFGE and CAAT was 100%.

2 Kennewicki is no longer recognized as a separate serovar from Pomona.[7]

3 NA = Not applicable for MLST since the species is not *L. interrogans* or *L. kirschneri*.

**Figure 1:**
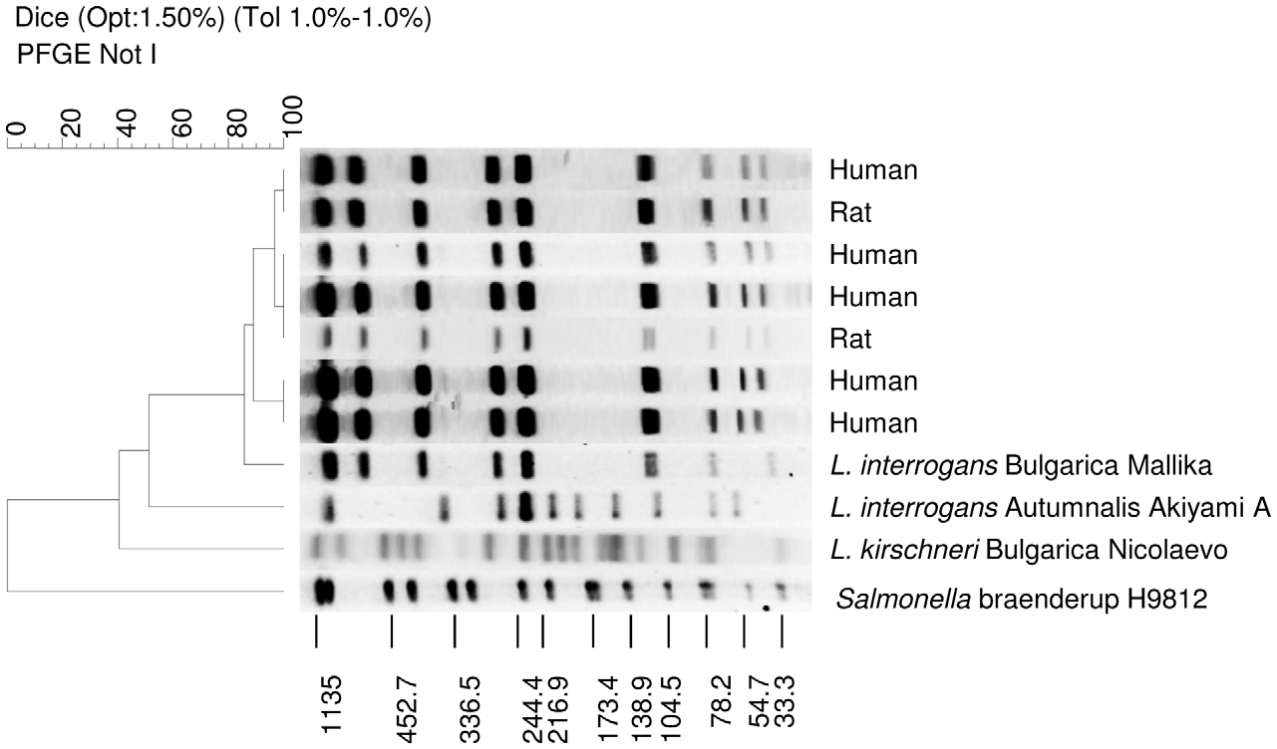
Selected PFGE patterns of isolates collected from humans and rats in Thailand along with two reference strains of serovar Bulgarica showing two different species and reference serovar Autumnalis. *Salmonella* Braenderup H9812 is shown as the size standard.

Although some serovars, such as Icterohaemorrhagiae/Copenhageni, were found to occur in most regions included in this study, there were some unique differences in geographic distribution of serovars. Among both rat and human isolates from Thailand, 87% (n = 27) were identified as *L. interrogans* serovar Bulgarica (Figure 1). In Brazil, 39% (n = 16) of isolates from dogs, swine and cattle were serovar Canicola. Six isolates (14%) from Brazil are being investigated as a new serovar and all were isolated from capybaras. However, serovar Icterohaemorrhagiae/Copenhageni was the most prevalent serovar isolated from human patients in Brazil (Table 1).

**Figure 2:**
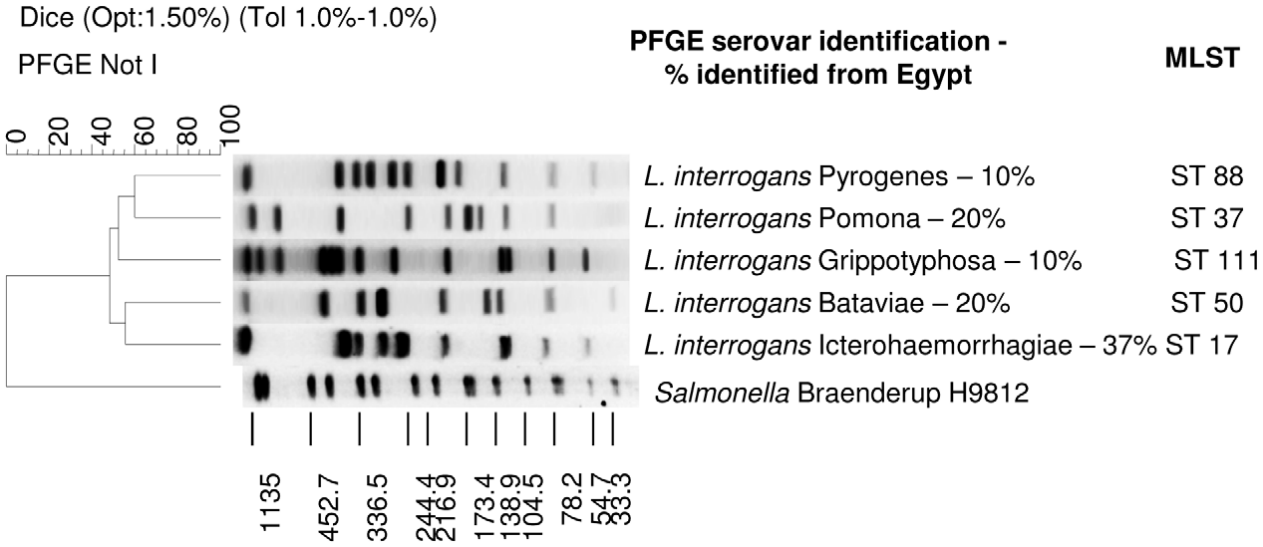
Representative PFGE patterns and MLST types from isolates identified from humans in Egypt and the proportions of each serovar identified among all Egyptian isolates. *Salmonella* Braenderup H9812 is shown as the size standard.

**Figure 3:**
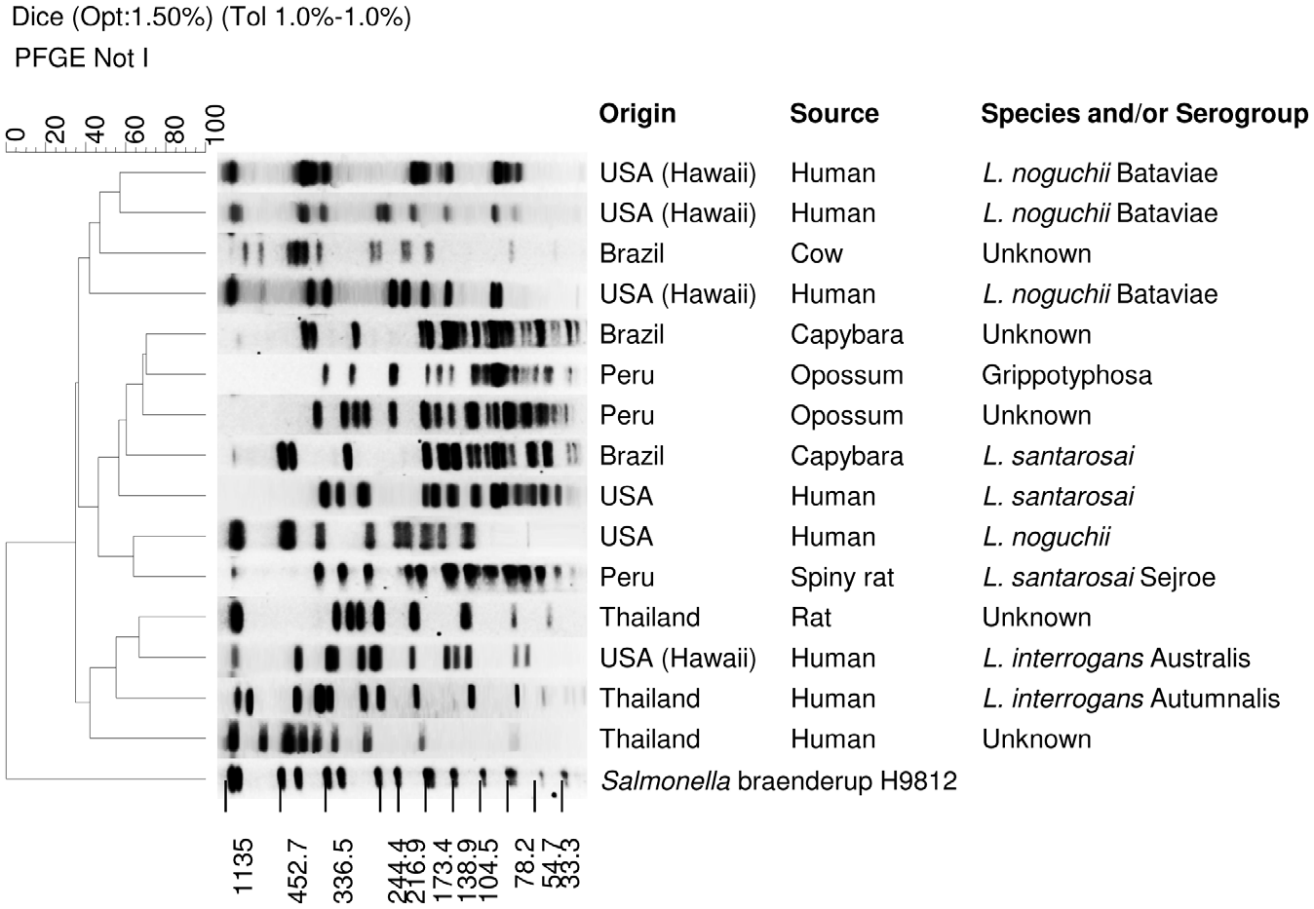
PFGE patterns of unidentified, potentially new serovars. *Salmonella* Braenderup H9812 is shown as the size standard.

The most common serovar identified from rats in Peru was Icterohaemorrhagiae/Copenhageni (45%, n = 10), but a recently described species (*L. licerasiae*)[5] isolated from both humans and rats made up 41% of the Peruvian isolates. Human isolates from Egypt were more diverse; serovars Bataviae, Grippotyphosa (*L. interrogans*), Icterohaemorrhagiae/Copenhageni, Pyrogenes and Pomona were identified (Figure 2). The majority of isolates from the United States were submitted from Hawaii, and among these, there are four novel fingerprint patterns by PFGE. Forty-one percent (n = 17) of the Hawaiian isolates make up one unknown pattern that is awaiting confirmation of new serovar status within *L. interrogans* (species confirmed by 16S rRNA gene sequencing). An additional 10% (n = 4) may represent three new serovars of *L. noguchii* (species confirmed by DNA hybridization) (Figure 3, Table 1). Four isolates from Hawaii were identified as closely related to most of the serovars in the Ballum serogroup; reference isolates for serovars Ballum, Castellonis, Guangdong, Arborea, and Soccoestomes are all within three band differences or less from one another in PFGE patterns. Serovar Kenya, the only remaining member of serogroup Ballum, had a distinct pattern that showed greater than 10 band differences from the other reference serovars in serogroup Ballum. Therefore, these four clinical isolates from Hawaii could not be definitely identified to the serovar level without using an additional enzyme, such as SgrAI.[14]

CAAT was performed to validate the use of PFGE as a serovar identification tool. Representative isolates from each country were selected for CAAT analysis. CAAT was performed on 36 isolates identified by PFGE as serovars Canicola, Icterohaemorrhagiae/Copenhageni, Ballum (or related serovar from serogroup Ballum), Bulgarica (*L. interrogans*), Pomona, Bataviae, Pyrogenes, and Grippotyphosa (*L. interrogans*). The correlation between PFGE and CAAT was 100% (35/35) (Table 1). There was one isolate which could not be fully resolved to the serovar level by either method. This was an isolate from Hawaii that resembled multiple serovars in serogroup Ballum by PFGE. Serologically, the isolate was related to serovar Ballum by CAAT, but could not be definitively identified as serovar Ballum (12.5–25% titer remaining after absorption, greater than the 10% cut off for serovars considered to be the same). Additional reference sera were unavailable at this time and will need to be produced and tested by CAAT, and the PFGE method needs to be optimized with a second enzyme in order to differentiate between serovars of serogroup Ballum. CAAT was unable to distinguish between isolates of serovars Icterohaemorrhagiae and Copenhageni using our reference antisera.

Isolates designated as potential new serovars based on PFGE profiles could not be identified by CAAT and are currently being evaluated at another reference institution for final confirmation of new serovar status (Figure 3, Table 1).

MLST was also performed on 42 isolates as an additional molecular characterization tool and to evaluate strain phylogeny. Results are displayed in Table 1. Three isolates from Brazil were ST37, the same ST type as reference serovars Pomona and Canicola. One isolate from Thailand was of ST34, which is the same as researchers found in Thailand.[19] Another isolate from Thailand represented both a new ST type as well as a new PFGE pattern. Twelve isolates from Hawaii were of ST51, the same as reference serovar Australis. Eight additional isolates from Hawaii were ST17, which matched the ST type of reference serovars Copenhageni and Icterohaemorrhagiae. Isolates from Egypt were ST17 (n = 3); ST37 (n = 1); ST50 (n = 5), which matches reference serovar Bataviae; ST88 (n = 3), which matches our reference strain of serovar Pyrogenes but differs from the three Pyrogenes serovars in the public database (ST types 13, 37, and 49); and ST111 (n = 1), which also matches our reference strain of serovar Grippotyphosa but differs from three Grippotyphosa serovars in the public database (ST types 18, 62 and 68). Lastly, the isolate from Guyana and two isolates from Peru were of ST17, which matches serovars Copenhageni and Icterohaemorrhagiae.

## Discussion

Multiple molecular techniques have been applied to the characterization of *Leptospira* isolates; however most can only identify to the species level (FAFLP,[23] RFLP[24], 16S rRNA sequence analysis.[21]) Other molecular characterization methods can provide strain information (MLVA, MLST, RFLP, repetitive element PCR) but are often limited to a few species and are not appropriate for all pathogenic species.[19], [25]–[29] PFGE has been used to identify isolates to the serovar level.[13]–[15], [24], [30] This technique is applicable to all pathogenic species and can rapidly identify potential new serovars.

MLST is a powerful molecular tool that has been applied recently to characterize isolates of *Leptospira* from several geographical locations, notably including a large outbreak in Thailand, which appears to have resulted largely from the expansion of a single clone (ST34).[19] However, MLST does not always correlate with the serovar. For example, serovars Pomona and Canicola share the same ST type (ST37) but are distinguishable by PFGE. Many of the isolates from Hawaii were characterized as ST51, the same ST type as serovar Australis; however the PFGE pattern and CAAT methods are more discriminatory for these isolates. Serovars Pyrogenes and Grippotyphosa, on the other hand, have multiple ST types for the same serovar.

Moreover, for a number of reasons, MLST is not generally applicable to all *Leptospira* spp. MLST has been applied in different locations, using different genes.[19], [31] Until the optimum set of sequences for MLST has been determined by examination of isolates with a global and historical distribution, and the scheme is applicable to all pathogenic species, [32] PFGE will remain the most widely applicable molecular characterization method. PFGE is also able to detect chromosomal rearrangements, whereas MLST in general is more useful for strain phylogeny. [33]

We presented the results of serovar identification for 175 clinical isolates worldwide. Isolates from humans as well as a wide range of animal hosts were analyzed. Although many common serovars were identified as expected, there were a large number of potential new serovars identified as well as isolates that require further investigation. Among the common serovars, Icterohaemorrhagiae/Copenhageni appeared to be the most prevalent in the majority of the countries, but regional differences in serovar distribution were apparent. *L. interrogans* serovar Bulgarica was the most prevalent serovar among human isolates in Thailand. Although there is a 3-band difference between the clinical isolates and the reference isolate, they are identified as the same serovar, using Tenover's criteria for strain typing. [34] These isolates were ST34 by MLST, the same sequence type found by Thaipadungpanit *et al*. in their paper describing the MLST method.[19] However, ST34 isolates in their study were identified as serovar Autumnalis, although the ST34 isolates in our study are different from serovar Autumnalis (Figure 1). Interestingly, ST34 isolates differ by only one out of seven alleles compared to *L. interrogans* serovar Bulgarica by MLST; whereas they differ by all seven alleles compared to *L. interrogans* serovar Autumnalis strain Akiyami A.

In this study, a subset of serovar identifications obtained by PFGE were validated by CAAT, the reference method for serovar identification. PFGE is a useful tool for serovar identification of clinical isolates and has the ability to facilitate recognition of potential new serovars with the advantages of a simpler, more standardized method than CAAT. Although our PFGE database does not yet contain all serovars (currently contains approximately 95% of all known serovars), it does allow us to identify rapidly the most common serovars. Continued use of PFGE to evaluate serovar identities will allow limited CAAT resources to be devoted to identification of isolates that cannot be identified readily by PFGE and to definitive characterization of new serovars. The use of PFGE can therefore aid in epidemiological studies and contribute to public health practices in order to decrease illnesses and outbreaks associated with leptospirosis.

## Supporting Information

Figure S1 Dendrogram of PFGE patterns comparing all clinical isolates used in this study.
(0.07 MB DOC)

## References

[1] World Health Organization (1999). Leptospirosis worldwide, 1999.. Weekly Epidemiological Record.

[2] Babudieri B (1958). Animal reservoirs of leptospirosis.. Annals of the New York Academy of Sciences.

[3] Levett PN (2001). Leptospirosis.. Clinical Microbiology Reviews.

[4] Bharti AR, Nally JE, Ricaldi JN, Matthias MA, Diaz MM (2003). Leptospirosis: a zoonotic disease of global importance.. Lancet Infectious Diseases.

[5] Matthias MA, Ricaldi JN, Cespedes M, Diaz MM, Galloway RL (2008). Human leptospirosis caused by a new, antigenically unique *Leptospira* associated with a *Rattus* species reservoir in the Peruvian Amazon.. PLoS Negl Trop Dis.

[6] Slack AT, Kalambaheti T, Symonds ML, Dohnt MF, Galloway RL (2008). *Leptospira wolffii* sp. nov., isolated from a human with suspected leptospirosis in Thailand.. Int J Syst Evol Microbiol.

[7] Kmety E, Dikken H (1993). Classification of the species *Leptospira interrogans* and history of its serovars..

[8] Mgode GF, Machang'u RS, Goris MG, Engelbert M, Sondij S (2006). New *Leptospira* serovar Sokoine of serogroup Icterohaemorrhagiae from cattle in Tanzania.. Int J Syst Evol Microbiol.

[9] Rossetti CA, Liem M, Samartino LE, Hartskeerl RA (2005). Buenos Aires, a new *Leptospira* serovar of serogroup Djasiman, isolated from an aborted dog fetus in Argentina.. Vet Microbiol.

[10] Valverde Mde L, Ramirez JM, Oca LG, Goris MG, Ahmed N (2008). Arenal, a new *Leptospira* serovar of serogroup Javanica, isolated from a patient in Costa Rica.. Infect Genet Evol.

[11] Faine S, Adler B, Bolin C, Perolat P (1999). *Leptospira* and leptospirosis..

[12] Galloway RL, Levett PN (2008). Evaluation of a modified pulsed-field gel electrophoresis approach for the identification of *Leptospira* serovars.. Am J Trop Med Hyg.

[13] Naigowit P, Charoenchai S, Biaklang M, Seena U, Wangroongsarb P (2007). Identification of clinical isolates of *Leptospira* spp by pulsed field gel-electrophoresis and microscopic agglutination test.. Southeast Asian J Trop Med Public Health.

[14] Herrmann JL, Bellenger E, Perolat P, Baranton G, Saint Girons I (1992). Pulsed-field gel electrophoresis of NotI digests of leptospiral DNA: a new rapid method of serovar identification.. J Clin Microbiol.

[15] Romero EC, Blanco RM, Galloway RL (2009). Application of pulsed-field gel electrophoresis for the discrimination of leptospiral isolates in Brazil.. Lett Appl Microbiol.

[16] Brenner DJ, Kaufmann AF, Sulzer KR, Steigerwalt AG, Rogers FC (1999). Further determination of DNA relatedness between serogroups and serovars in the family Leptospiraceae with a proposal for *Leptospira alexanderi* sp. nov. and four new *Leptospira* genomospecies.. Int J Syst Bacteriol.

[17] Hunter SB, Vauterin P, Lambert-Fair MA, Van Duyne MS, Kubota K (2005). Establishment of a universal size standard strain for use with the PulseNet standardized pulsed-field gel electrophoresis protocols: converting the national databases to the new size standard.. J Clin Microbiol.

[18] Majed Z, Bellenger E, Postic D, Pourcel C, Baranton G (2005). Identification of variable-number tandem-repeat loci in *Leptospira interrogans* sensu stricto.. J Clin Microbiol.

[19] Thaipadungpanit J, Wuthiekanun V, Chierakul W, Smythe LD, Petkanchanapong W (2007). A Dominant Clone of *Leptospira interrogans* Associated with an Outbreak of Human Leptospirosis in Thailand.. PLoS Negl Trop Dis.

[20] Dikken H, Kmety E, Bergan T, Norris JR (1978). Serological typing methods of leptospires.. Methods in Microbiology.

[21] Morey RE, Galloway RL, Bragg SL, Steigerwalt AG, Mayer LW (2006). Species-specific identification of Leptospiraceae by 16S rRNA gene sequencing.. J Clin Microbiol.

[22] Mandel M, Igambi L, Bergendahl J, Dodson ML, Scheltgen E (1970). Correlation of melting temperature and cesium chloride buoyant density of bacterial deoxyribonucleic acid.. J Bacteriol.

[23] Vijayachari P, Ahmed N, Sugunan AP, Ghousunnissa S, Rao KR (2004). Use of fluorescent amplified fragment length polymorphism for molecular epidemiology of leptospirosis in India.. J Clin Microbiol.

[24] Turk N, Milas Z, Mojcec V, Ruzic-Sabljic E, Staresina V (2009). Molecular analysis of *Leptospira* spp. isolated from humans by restriction fragment length polymorphism, real-time PCR and pulsed-field gel electrophoresis.. FEMS Microbiol Lett.

[25] Romero EC, Yasuda PH (2006). Molecular characterization of *Leptospira* sp. strains isolated from human subjects in Sao Paulo, Brazil using a polymerase chain reaction-based assay: a public health tool.. Mem Inst Oswaldo Cruz.

[26] Bourhy P, Collet L, Clement S, Huerre M, Ave P (2010). Isolation and characterization of new *Leptospira* genotypes from patients in Mayotte (Indian Ocean).. PLoS Negl Trop Dis.

[27] Zakeri S, Sepahian N, Afsharpad M, Esfandiari B, Ziapour P (2010). Molecular epidemiology of leptospirosis in northern Iran by nested polymerase chain reaction/restriction fragment length polymorphism and sequencing methods.. Am J Trop Med Hyg.

[28] Ellis WA, Montgomery JM, Thiermann AB (1991). Restriction endonuclease analysis as a taxonomic tool in the study of pig isolates belonging to the Australis serogroup of *Leptospira interrogans*.. J Clin Microbiol.

[29] Slack AT, Dohnt MF, Symonds ML, Smythe LD (2005). Development of a Multiple-Locus Variable Number of Tandem Repeat Analysis (MLVA) for *Leptospira interrogans* and its application to *Leptospira interrogans* serovar Australis isolates from Far North Queensland, Australia.. Ann Clin Microbiol Antimicrob.

[30] Ciceroni L, Ciarrocchi S, Ciervo A, Petrucca A, Pinto A (2002). Differentiation of leptospires of the serogroup Pomona by monoclonal antibodies, pulsed-field gel electrophoresis and arbitrarily primed polymerase chain reaction.. Res Microbiol.

[31] Ahmed N, Devi SM, Valverde Mde L, Vijayachari P, Machang'u RS (2006). Multilocus sequence typing method for identification and genotypic classification of pathogenic *Leptospira* species.. Ann Clin Microbiol Antimicrob.

[32] Levett PN (2007). Sequence-based typing of *Leptospira*: epidemiology in the genomic era.. PLoS Negl Trop Dis.

[33] Vimont S, Mnif B, Fevre C, Brisse S (2008). Comparison of PFGE and multilocus sequence typing for analysis of *Klebsiella pneumoniae* isolates.. J Med Microbiol.

[34] Tenover FC, Arbeit RD, Goering RV, Mickelsen PA, Murray BE (1995). Interpreting chromosomal DNA restriction patterns produced by pulsed-field gel electrophoresis: criteria for bacterial strain typing.. J Clin Microbiol.

