# Supplementary material for: Application and Validation of PFGE for Serovar Identification of Leptospira Clinical Isolates
Source: PLoS Negl Trop Dis. 2010 Sep 14;4(9):e824. doi: 10.1371/journal.pntd.0000824 (PMC2939049; doi:10.1371/journal.pntd.0000824)
Supplement: Supplementary file 1 [file pntd.0000824.s001.doc]

Dice (Opt:1.50%) (Tol 1.0%-1.0%) (H>0.0% S>0.0%) [0.0%-100.0%]
PFGE Not I

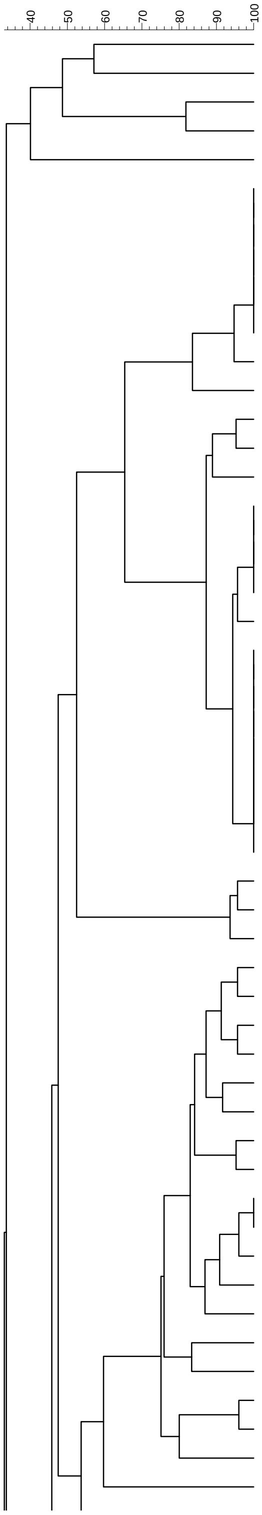

.1993005606	..noguchii (DNA hyb), k.	Bataviae		Human	USA
.2007001578	..noguchii (DNA hyb), k.	Bataviae	Waimea ?	Human	USA
.2001034031	..noguchii	Bataviae	unknown	Human	USA
.2004000358	..noguchii	Bataviae	unknown	Human	USA
.lepto0096	..unknown	unknown	unknown	Cow	Brazil
.2006006989	..interrogans	Pomona	Pomona	Human	Egypt
.2006006983	..interrogans	Pomona	Pomona	Human	Egypt
.2006006962	..interrogans	Pomona	Pomona	Human	Egypt
.2006006969	..interrogans	Pomona	Pomona	Human	Egypt
.2006006968	..interrogans	Pomona	Pomona	Human	Egypt
.2006006964	..interrogans	Pomona	Pomona	Human	Egypt
.2006006988	..interrogans	Pomona	Pomona	Human	Egypt
.lepto0085	..interrogans	Pomona	Kennewicki	Swine	Brazil
.lepto0113	..interrogans	Canicola	Canicola	Dog	Brazil
.lepto0105	..interrogans	Canicola	Canicola	Dog	Brazil
.lepto0032	..interrogans	Canicola	Portlandvere	Swine	Brazil
.lepto0093	..interrogans	Canicola	Canicola	Dog	Brazil
.lepto0095	..interrogans	Canicola	Canicola	Swine	Brazil
.lepto0097	..interrogans	Canicola	Canicola	Cow	Brazil
.lepto0086	..interrogans	Canicola	Canicola	Dog	Brazil
.lepto0094	..interrogans	Canicola	Canicola	Dog	Brazil
.lepto0435	..interrogans	Canicola	Canicola	Human	Brazil
.lepto0114	..interrogans	Canicola	Canicola	Dog	Brazil
.lepto0115	..interrogans	Canicola	Canicola	Dog	Brazil
.lepto0116	..interrogans	Canicola	Canicola	Dog	Brazil
.lepto0031	..interrogans	Canicola	Canicola	Dog	Brazil
.lepto0106	..interrogans	Canicola	Canicola	Dog	Brazil
.lepto0103	..interrogans	Canicola	Canicola	Dog	Brazil
.lepto0434	..interrogans	Canicola	Canicola	Human	Brazil
.2006006973	..interrogans	Pyrogenes	Pyrogenes	Human	Egypt
.2006006960	..interrogans	Pyrogenes	Pyrogenes	Human	Egypt
.2006006956	..interrogans	Pyrogenes	Pyrogenes	Human	Egypt
.2005005624	..interrogans	Australis	New	Human	USA
.2002000621	..interrogans	Australis	New	Human	USA
.2002000624	..interrogans	Australis	New	Human	USA
.2004018171	..interrogans	Australis	New	Human	USA
.2002000626	..interrogans	Australis	New	Human	USA
.2002000627	..interrogans	Australis	New	Human	USA
.2002000631	..interrogans	Australis	New	Human	USA
.2002000625	..interrogans	Australis	New	Human	USA
.2003000735	..interrogans	Australis	New	Human	USA
.2003000972	..interrogans	Australis	New	Human	USA
.2000010763	..interrogans	Australis	New	Human	USA
.2002000628	..interrogans	Australis	New	Human	USA
.2000010762	..interrogans	Australis	New	Human	USA
.2002000629	..interrogans	Australis	New	Human	USA
.2002000632	..interrogans	Australis	New	Human	USA
.2002000623	..interrogans	Australis	New	Human	USA
.2002000633	..interrogans	Australis	New	Human	USA
.2002000630	..interrogans	Australis	New	Human	USA
.lepto0034	..interrogans	Autumnalis	New	Human	Thaila
[illegible]	[illegible]	[illegible]	[illegible]	[illegible]	[illegible]

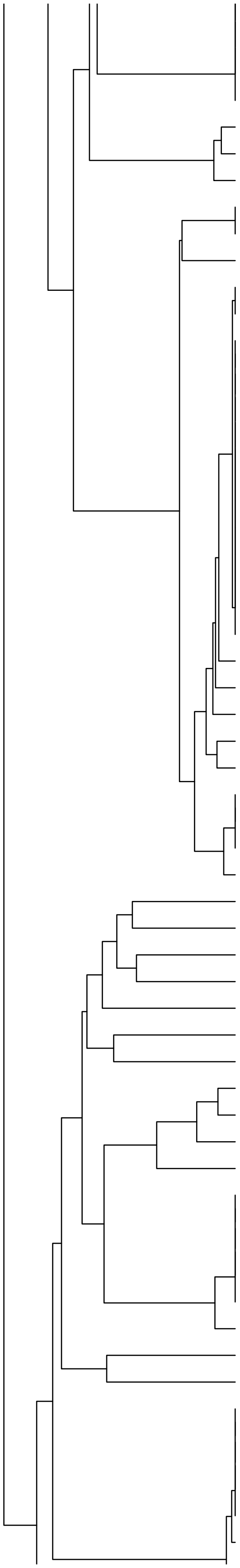

.2006006976	..interrogans	Bataviae	Bataviae	Human	Egypt
.2006006974	..interrogans	Bataviae	Bataviae	Human	Egypt
.2006006975	..interrogans	Bataviae	Bataviae	Human	Egypt
.2006006959	..interrogans	Bataviae	Bataviae	Human	Egypt
.2006006970	..interrogans	Grippotyphosa	Grippotyphosa	.Human	Egypt
.2006006986	..interrogans	Grippotyphosa	Grippotyphosa	Human	Egypt
.2006006971	..interrogans	Grippotyphosa	Grippotyphosa	.Human	Egypt
.lepto0072	..interrogans	Autumnalis	Bulgarica	Human	Thaila
.lepto0073	..interrogans	Autumnalis	Bulgarica	Human	Thaila
.lepto0035	..interrogans	Autumnalis	Bulgarica	Human	Thaila
.lepto0067	..interrogans	Autumnalis	Bulgarica	Human	Thaila
.lepto0098	..interrogans	Autumnalis	Bulgarica	Human	Thaila
.lepto0050	..interrogans	Autumnalis	Bulgarica	Human	Thaila
.lepto0051	..interrogans	Autumnalis	Bulgarica	Human	Thaila
.lepto0029	..interrogans	Autumnalis	Bulgarica	Human	Thaila
.lepto0053	..interrogans	Autumnalis	Bulgarica	Human	Thaila
.lepto0030	..interrogans	Autumnalis	Bulgarica	Human	Thaila
.lepto0054	..interrogans	Autumnalis	Bulgarica	Human	Thaila
.lepto0049	..interrogans	Autumnalis	Bulgarica	Human	Thaila
.lepto0112	..interrogans	Autumnalis	Bulgarica	Human	Thaila
.lepto0066	..interrogans	Autumnalis	Bulgarica	Human	Thaila
.lepto0055	..interrogans	Autumnalis	Bulgarica	Rat	Thaila
.lepto0056	..interrogans	Autumnalis	Bulgarica	Rat	Thaila
.lepto0037	..interrogans	Autumnalis	Bulgarica	Rat	Thaila
.lepto0111	..interrogans	Autumnalis	Bulgarica	Human	Thaila
.lepto0078	..interrogans	Autumnalis	Bulgarica	Human	Thaila
.lepto0052	..interrogans	Autumnalis	Bulgarica	Human	Thaila
.lepto0108	..interrogans	Autumnalis	Bulgarica	Human	Thaila
.lepto0068	..interrogans	Autumnalis	Bulgarica	Human	Thaila
.lepto0027	..interrogans	Autumnalis	Bulgarica	Human	Thaila
.lepto0026	..interrogans	Autumnalis	Bulgarica	Human	Thaila
.lepto0028	..interrogans	Autumnalis	Bulgarica	Human	Thaila
.lepto0025	..interrogans	Autumnalis	Bulgarica	Human	Thaila
.lepto0248	..unknown	Grippotyphosa	unknown	Didephis Marsupialis	Peru
.lepto0138	..unknown	unknown	unknown	Capybara	Brazil
.lepto0191	..santarosai	No known serogroup	unknown	Philander spp.	Peru
.lepto0139	..biflexa			Rattus norvegicus	Brazil
.2000027870	.santarosai	Hebdomadis	unknown	Human	USA
.lepto0197	..santarosai	Sejroe	unknown	Proechimys spp.	Peru
.2000030832	.santarosai 99.93%	Hebdomadis		Human	USA
.2002000620	..borgpetersenii	Ballum	Ballum/Castellonis	Human	USA
.2002020829	..borgpetersenii	Ballum	Ballum/Castellonis	Human	USA
.2004018167	..borgpetersenii	Ballum	Ballum/Castellonis	Human	USA
.2004018169	..borgpetersenii	Ballum	Ballum/Castellonis	Human	USA
.lepto0033	..santarosai		New	Capybara	Brazil
.lepto0036	..santarosai		New	Capybara	Brazil
.lepto0137	..santarosai		New	Capybara	Brazil
.lepto0107	..santarosai		New	Capybara	Brazil
.lepto0091	..santarosai		New	Capybaras	Brazil
.lepto0117	..santarosai		New	Capybara	Brazil
.lepto0270	.broomii			.Human	Franc
.lepto0043	..broomii			.Human	Denm
.lepto0176	..licerasiae			Rattus norvegicus	Peru
.lepto0177	..licerasiae			Rattus norvegicus	Peru
.lepto0178	..licerasiae			Rattus norvegicus	Peru
.lepto0180	..licerasiae			Rattus norvegicus	Peru
.lepto0182	..licerasiae			Human	Peru
.lepto0181	..licerasiae			Rattus Rattus	Peru
[illegible]	[illegible]			[illegible]	[illegible]
